# Photocatalysis of Cr- and Fe-Doped CeO_2_ Nanoparticles to Selective Oxidation of 5-Hydroxymethylfurfural

**DOI:** 10.3390/nano13010044

**Published:** 2022-12-22

**Authors:** Jeong-Woo Nam, Vy Ngoc Pham, Jeong Min Ha, Minjeong Shin, Hangil Lee, Young-Sang Youn

**Affiliations:** 1 Department of Chemistry, Yeungnam University, Daehak-ro 280, Gyeongsan, Gyeongbuk 38541, Republic of Korea; 2 Department of Chemistry, Sookmyung Women’s University, Seoul 04310, Republic of Korea; 3 School of Chemistry and Energy, Sungshin Women’s University, 55, Dobong-ro 76 ga-gil, Gangbuk-gu, Seoul 01133, Republic of Korea

**Keywords:** transition metal-doped CeO_2_ nanoparticles, oxygen vacancy, photocatalytic property, 5-hydroxymethylfurfural

## Abstract

Oxygen vacancies (*V_o_*) present in CeO_2_ nanoparticles (NPs) can effectively boost their photocatalytic activity under ultraviolet (UV) light. To improve photocatalytic performance, Cr- and Fe-doped CeO_2_ NPs with increased *V_o_* were prepared using a simple method of doping Cr and Fe ions into CeO_2_ NPs, which was confirmed by an in-depth analysis of the structural and electronic changes. Through photocatalytic degradation (PCD) experiments with 5-hydroxymethylfurfural (HMF), we found that the PCD rates of the two doped CeO_2_ NPs were faster than that of the CeO_2_ NPs. In addition, the conversion of HMF to 2,5-furandicarboxylic acid (FDCA) using the doped CeO_2_ NPs occurred only through the mechanism of the selective oxidation to 5-hydroxymethyl-2-furancarboxylic acid (HMFCA), exhibiting better efficiency than using CeO_2_ NPs.

## 1. Introduction

Over the last few decades, metal oxides, such as CeO_2_, ZnO, and TiO_2_, have been studied for applications in various commercial fields [1,2,3]. Among metal oxides, CeO_2_ is considerably attractive because it has the unique characteristics of a reversible valence change between Ce^4+^ and Ce^3+^ and oxygen vacancies (*V_o_*) [4], leading to the engineering of *V_o_* to improve its photocatalytic performance [5,6,7]. With the tunable photocatalytic properties of CeO_2_, photocatalysis by its action has been highlighted as a promising method for solving energy issues with plentiful sunlight [8,9].

**Scheme 1:**
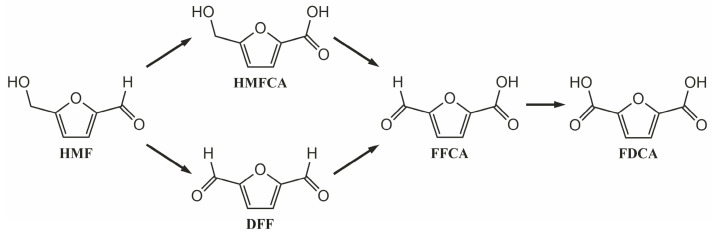
Two reaction pathways for the conversion from HMF to FDCA.

Among various energy problems, the depletion of petroleum-based fuels is primary tasks to be solved because they directly affect the welfare of humanity. In this regard, cheap and abundant biomass is being considered as a potential alternative to petroleum-based fuels. The effective utilization of biomass can play a crucial role in solving the current energy crisis [10,11]. To this end, a facile strategy involves the oxidation of biomass and its derivatives using CeO_2_ nanoparticles (NPs) as photocatalysts, and numerous efforts have been made to understand the mechanism of such reactions for various practical applications [12,13,14]. One of the most widely explored molecules as a biomass platform compound is 5-hydroxymethylfurfural (HMF), which is capable of being selectively oxidized through its two different functional groups: an aldehyde and alcohol [15,16]. To be precise, HMF can be converted to 2,5-furandicarboxylic acid (FDCA) through the production of 5-formyl-2-furancarboxylic acid (FFCA) by two distinct pathways; one is through 5-hydroxymethyl-2-furancarboxylic acid (HMFCA) by the preferential oxidation of the aldehyde group of HMF and the other is via 2,5-diformethylfuran (DFF) by that of its alcohol group (Scheme 1) [12,17,18]. HMFCA and FDCA produced by HMF are considered suitable replacements of furanic polyester and phthalic acid obtained from fossil resources, respectively [19,20,21]. In particular, FDCA is recognized by the U.S. Department of Energy as one of the top 12 bio-based chemicals [22,23]. Therefore, the selective production of HMFCA and FDCA from HMF and the increase in conversion efficiency using CeO_2_ NPs as photocatalysts have become a research hotspot [24].

In pursuit of highly selective and efficient production of HMFCA/FDCA, we introduced simple transition metal (TM)-ion doping into CeO_2_ NPs. It is known that the amount of *V*_o_ in CeO_2_ NPs increases with the modification of the CeO_2_ surface from Cr- and Fe-ion doping [9,25]. Therefore, we explored the photocatalytic activity of three distinct CeO_2_, Cr-doped CeO_2_ (Cr@CeO_2_), and Fe-doped CeO_2_ (Fe@CeO_2_) NPs through photocatalytic experiments with HMF. In addition, the structural and electronic changes between CeO_2_ and the doped CeO_2_ NPs were thoroughly analyzed using a combination of high-resolution transmission electron microscopy (HRTEM), scanning transmission electron microscopy with energy dispersive X-ray spectroscopy (STEM-EDS), X-ray diffraction (XRD), Raman spectroscopy, ultraviolet-visible diffuse reflectance spectroscopy (UV-Vis DRS), and X-ray absorption spectroscopy (XAS).

## 2. Materials and Methods

### 2.1. Chemicals and NP Preparation

CeO_2_ NPs were synthesized using a modified thermal method [26]. CeCl_3_∙7H_2_O (0.2 g; Sigma-Aldrich, St. Louis, MO, USA, 99.9%) and PVP (0.3 g; Sigma-Aldrich) were dissolved in 40 mL of deionized water under vigorous magnetic stirring. Then, 1 mL of formamide (Sigma-Aldrich, 99.9%) and 0.2 mL of H_2_O_2_ (Sigma-Aldrich) were added to the solution under continuous stirring at 90 rpm for 1 h. The as-formed yellow solution was treated by adding KOH (Sigma-Aldrich) until the pH was 13.0 and then held at that pH for 3 h. Next, the solution was transferred into a Teflon-lined autoclave and heated for 9 h at 220 °C. The resulting CeO_2_ NPs were filtered and washed with double-distilled water (DDW) to remove any impurities. The products (CeO_2_ NPs) were then washed with absolute ethanol and dried for 48 h at 95 °C. After fabricating the CeO_2_ NPs [26,27], two different TM ions (TM nitrate *n*-hydrate: Cr(NO_3_)_3_∙9H_2_O and Fe(NO_3_)_3_∙6H_2_O, Sigma-Aldrich, 99%) were added to the CeO_2_ NPs at 90 °C with moderate stirring until a homogeneous and transparent mixture was formed (approximately 3 h). The obtained solution was then heated at 220 °C for 9 h. The resulting TM@CeO_2_ NPs were filtered and washed with DDW to remove any impurities, resulting in TM@CeO_2_ NPs with 5 wt% Cr^3+^ or Fe^3+^ ions. The photocatalytic properties of CeO_2_, Cr@CeO_2_, and Fe@CeO_2_ NPs were determined using HMF (Sigma-Aldrich, 99%).

### 2.2. Characterization

The morphology of CeO_2_, Cr@CeO_2_, and Fe@CeO_2_ NPs was analyzed by HRTEM (JEM-ARM200CF, JEOL Ltd., Tokyo, Japan) using an accelerating voltage of 200 kV. In addition, the distribution of the constituent elements within the NPs at the nanoscale was mapped using STEM-EDS (JED-2300T, JEOL Ltd., Japan). The XRD data were recorded in the range 20–100° in a scanning step of 0.02° for 0.3 s using a MiniFlex600 system (Rigaku, Tokyo, Japan) with Cu K_α_ radiation operated at 15 mA and 40 kV. Crystallite sizes and lattice parameters were calculated by Pawley refinement of the corresponding diffraction patterns using TOPAS software (Version 4.2, Bruker, Rheinstetten, Germany). Raman spectra were obtained using an XploRA Raman spectrometer (HORIBA, Kyoto, Japan) with a diode-pumped solid-state laser of 532 nm wavelength operating at 10 mW. The UV-Vis DRS experiments were performed on a UV-2600i UV-Vis spectrophotometer (Shimadzu, Kyoto, Japan) equipped with integrated spheres. To compare the electronic structure of CeO_2_ and TM@CeO_2_ NPs, we obtained Ce *M*-edge, O *K*-edge, Cr *L*-edge, and Fe *L*-edge spectra using XAS in the 8A1 beamline at the Pohang Accelerator Laboratory with a PHI-3057 electron analyzer (Physical Electronics, Chanhassen, MN, USA) under the base pressure of 2.0 × 10^−9^ Torr.

### 2.3. Photocatalytic Experiments

To evaluate the photocatalytic degradation (PCD) activity, suspensions of CeO_2_, Cr@CeO_2_, and Fe@CeO_2_ NPs (0.5 g/L) containing HMF (25 mM) were stirred in the dark for 2 h until adsorption equilibrium was attained. A 30 mL volume reactor with a solution containing each NP was placed in front of a blue light-emitting diode (LED) (λ = 365 nm, output power = 6 W; Thorlabs, Newton, NJ, USA) at a distance of 10 cm and magnetically stirred at 80 rpm with and without 5,5-dimethyl-1-pyrroline N-oxide (DMPO; 50 mM). At the distance of 10 cm from the blue LED along the irradiation axis, the light intensity was determined as 10 ± 0.7 mW/cm^2^. The HMF oxidation products were analyzed using high-performance liquid chromatography-mass spectrometry (HPLC-MS; ULTIMATE 3000 RSLC SYSTEM, Thermo Fisher Scientific and Q-EXACTIVE ORBITRAP PLUS MS, Thermo Fisher Scientific, Waltham, MA, USA).

## 3. Results

### 3.1. Characterization of TM@CeO_2_ NPs

**Figure 1:**
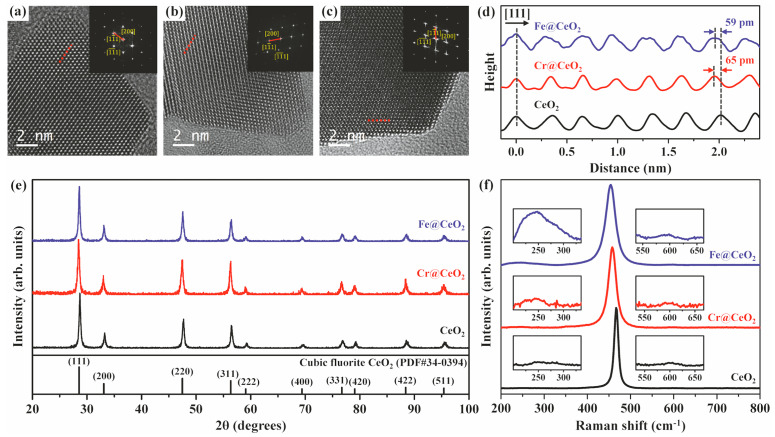
HRTEM images of (**a**) CeO_2_, (**b**) Cr@CeO_2_, and (**c**) Fe@CeO_2_ NPs. Insets: The corresponding FFT data. (**d**) Line profile of the red line shown in (**a**–**c**) for the corresponding NPs. The decrease in the interlayer d-spacings of the [111] plane for Cr@CeO_2_ and Fe@CeO_2_ NPs is measured as 65 and 59 pm on the six lattice fringes compared to that for CeO_2_ NPs, indicating 10.8 and 9.8 pm less per lattice fringe, respectively. (**e**) XRD and (**f**) Raman spectra of CeO_2_, Cr@CeO_2_, and Fe@CeO_2_ NPs. Bottom panel in (**e**): Diffraction pattern of cubic fluorite CeO_2_ (PDF#34-0394) with Miller indices extracted from the PDF-2 database.

HRTEM images of CeO_2_, Cr@CeO_2_, and Fe@CeO_2_ NPs are shown in Figure 1a–c and Figure S1. As shown in the HRTEM images and the inserted fast Fourier transform (FFT) forms, there is no principal structural change between the three NPs (Figure 1a–c). In addition, on the basis of the STEM images and elemental distribution in the STEM-EDS data (Figure S2), we confirm that the TM ions are homogeneously distributed in the TM@CeO_2_ NPs. To obtain additional structural information on the particle regions by closer inspection, the line profiles were analyzed at regular intervals on the sub-nanometer scale for the three NPs, as shown in Figure 1d. Compared with CeO_2_ NPs, Cr@CeO_2_ and Fe@CeO_2_ NPs exhibited a decrease of 10.8 and 9.8 pm per lattice fringe in the interlayer d-spacing of the [111] plane, respectively. In general, compared to CeO_2_ NPs, the smaller interlayer d-spacing in TM@CeO_2_ NPs reflects the higher density of *V*_o_ [25,28], suggesting the successful fabrication of highly defective TM@CeO_2_ NPs by Cr and Fe ion doping. Therefore, we can predict from the interlayer d-spacing values that the photocatalytic properties of the TM@CeO_2_ NPs will be better than those of the CeO_2_ NPs.

Next, XRD experiments were performed to investigate the changes in crystal structure of the TM@CeO_2_ NPs by doping. In addition, the crystallite sizes and lattice parameters were determined through Pawley refinement of the obtained XRD spectra. As shown in Figure 1e, all the XRD spectra correspond to the diffraction patterns associated with the cubic fluorite CeO_2_ crystal structure with the space group Fm-3m, indicating that no principal structural change occurred by TM ion doping, in accordance with the HRTEM results. Moreover, peak broadening for TM@CeO_2_ NPs compared to CeO_2_ NPs is not clearly recognized in the XRD spectra, which indicates that their grain sizes are similar [9]. The grains in nanoparticles are known to consist of crystallites [29]. Therefore, if we estimate the crystallite size from the XRD data, we can infer the grain size from the crystallite size, which is defined as the coherent diffraction domain. As shown in Table 1, we can clearly confirm that the crystallite sizes of the TM@CeO_2_ NPs are similar to those of the CeO_2_ NPs within an error range, indicating that the effect of the size of the NPs does not need to be considered in our results. Furthermore, the *a*-axis lattice parameter of the cubic fluorite CeO_2_ crystal structure for TM@CeO_2_ NPs is slightly smaller than that for CeO_2_ NPs, showing a trend similar to the results of the line profiles measured from the HRTEM images shown in Figure 1d. It can be inferred that the shrinkage of the lattice constant for TM@CeO_2_ NPs is due to the introduction of dopants into CeO_2_ NPs, leading to an increase in *V*_o_, as reported in the literature [30]. Therefore, we expect that the photocatalytic abilities of the TM@CeO_2_ NPs will be superior to those of the CeO_2_ NPs, in agreement with the HRTEM data.

Figure 1f shows the Raman spectra of CeO_2_, Cr@CeO_2_, and Fe@CeO_2_ NPs, where a strong band (F_2g_) appears at approximately 467 cm^−1^ due to the symmetric breathing mode of the oxygen ions coordinated with each Ce^4+^ ion in the cubic fluorite CeO_2_ structure [31]. Through the Raman profiles, we also confirm that TM ion doping into the CeO_2_ NPs barely influences the CeO_2_ structure. In addition to the F_2g_ peak, two weak bands related to the transverse acoustic mode and the non-degenerate longitudinal optical mode are observed at approximately 250 and 600 cm^−1^ in Figure 1f, respectively [32,33]. These two peaks for the TM@CeO_2_ NPs are stronger than those for the CeO_2_ NPs. According to the literature, these bands can be attributed to the presence of defects dominantly created by *V*_o_ [32,33]. In particular, the peak at approximately 600 cm^−1^ arises from *V*_o_ caused by the reduced Ce^3+^ ions [34]. Hence, we anticipate that TM@CeO_2_ NPs will have better photocatalytic properties than those of CeO_2_ NPs, owing to the increase in the number of defects attributed to *V*_o_.

### 3.2. Electronic Properties of TM@CeO_2_ NPs

**Figure 2:**
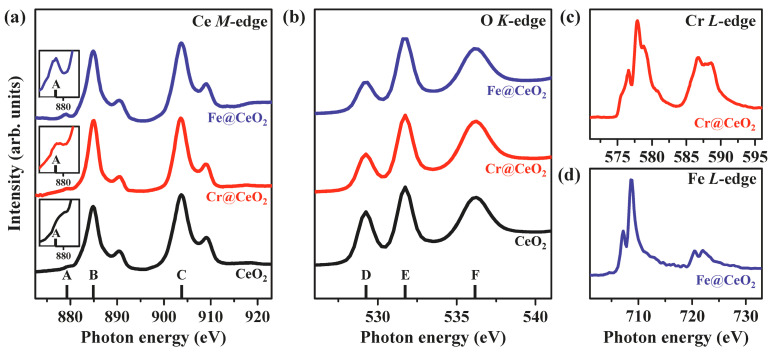
XAS profiles of (**a**) Ce *M*-edge, (**b**) O *K*-edge, (**c**) Cr *L*-edge, and (**d**) Fe *L*-edge for CeO_2_, Cr@CeO_2_, and Fe@CeO_2_ NPs.

The doping of CeO_2_ NPs with Cr or Fe ions changes the properties of the material at the nanoscale. To understand the implication of this process on the photocatalytic activity, a deeper understanding of the effect of the dopants on the change in electronic properties within the material is necessary. To investigate it, we performed UV-Vis DRS experiments to measure the band gap of the CeO_2_, Cr@CeO_2_, and Fe@CeO_2_ NPs. As shown in Figure S3, the band gap of CeO_2_ NPs was determined to be 3.26 eV, which is almost identical to the reported value of 3.25 eV [35]. Glancing the change in band gap by the dopants, the band gap of Cr@CeO_2_ and Fe@CeO_2_ NPs slightly increases to 3.32 and 3.34 eV, respectively, suggesting the modulation of the band gap of the CeO_2_ NPs by the dopants. To obtain information on the electronic structure of the unoccupied state at the NP surface, we acquired the XAS profiles of the Ce *M*-edge, O *K*-edge, Cr *L*-edge, and Fe *L*-edge for the CeO_2_, Cr@CeO_2_, and Fe@CeO_2_ NPs, as shown in Figure 2, where the spectra are normalized and overlaid vertically to identify changes in the electronic structure. In Figure 2a, the peaks corresponding to M_5_ (marked as B) and M_4_ (marked as C) are clearly observed, and additional satellite peaks are also found at higher photon energies for all NPs [36]. The B/C intensity ratio for the two TM@CeO_2_ NPs is slightly higher than that of the CeO_2_ NPs. This indicates that more Ce^3+^ states generated by the oxygen defect structure exist in TM@CeO_2_ NPs [37,38]. In addition, the pre-edge peak (marked as A) is directly related to the defect structure. As can be seen from the Ce *M*-edge spectra, the intensity of peak A for the two TM@CeO_2_ NPs is slightly higher than that for the CeO_2_ NPs. Therefore, the photocatalytic effects of the TM@CeO_2_ NPs containing more defects are expected to be better than that of CeO_2_ NPs. The peaks marked as D, E, and F in the O *K*-edge spectra (Figure 2b) correspond to transition-induced O 2p-Ce 4f, Ce 5d-e_g_, and Ce 5d-t_2g_ states, respectively [7,39]. Focusing on the relative intensity between peaks D and E, it can be seen that the D/E intensity ratio of the two TM@CeO_2_ NPs is lower than that of the CeO_2_ NPs. This indicates that the number of defects in TM@CeO_2_ NPs is greater than in the CeO_2_ NPs [40,41,42,43]. To confirm the oxidation state and electronic structure of the TMs formed on the surface of the TM@CeO_2_ NPs, we measured the Cr *L*-edge spectrum for Cr@CeO_2_ NPs and the Fe *L*-edge spectrum for Fe@CeO_2_ NPs (Figure 2c–d). From these spectra, we confirm that the Cr and Fe *L*-edge profiles show the shapes of typical Cr_2_O_3_ and Fe_2_O_3_ composition, respectively [44,45,46,47]. From the XAS data, we envision that the formation of chromium or iron oxides on the surface of the TM@CeO_2_ NPs can give rise to oxygen deficiencies, leading to the promotion of conversion of Ce^4+^ to Ce^3+^ ions to compensate for the charge valence. As a result, we propose that the concentration of *V*_o_ in the TM@CeO_2_ NPs is higher than that in the CeO_2_ NPs, resulting in an increase in photocatalytic performance.

### 3.3. Selective Production of HMFCA and High Conversion Efficiency to FDCA by Photolysis of HMF in the Presence of TM@CeO_2_ NPs

**Figure 3:**
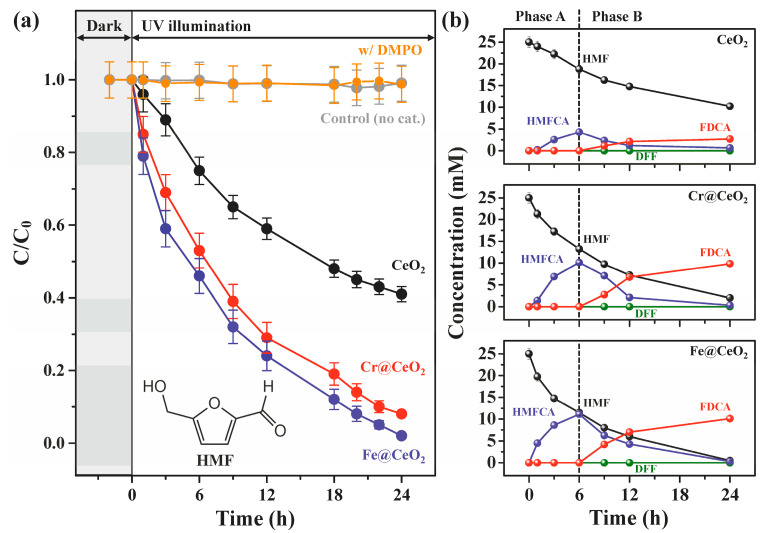
(**a**) PCD data with and without DMPO as a radical scavenger for the change in the relative amount of HMF and (**b**) quantitative analysis of products by photolysis of HMF under UV irradiation of 365 nm wavelength in the presence of CeO_2_, Cr@CeO_2_, and Fe@CeO_2_ NPs. C_0_ and C indicate the initial concentration and post-reaction concentration of HMF, respectively.

Maximizing the amount of *V*_o_ by doping without altering the principal structure of CeO_2_ NPs is one of the easiest and most effective strategies for improving the efficiency of the photocatalyst. To verify this, we investigated the photocatalytic activity of CeO_2_, Cr@CeO_2_, and Fe@CeO_2_ NPs in the PCD reaction of HMF. As shown in Figure 3a, the PCD reaction rates of HMF in the presence of the two TM@CeO_2_ NPs are higher than that of the CeO_2_ NPs. Table 2 presents the photocatalytic efficiency of the three NPs for the PCD reaction of HMF for 24 h, and the PCD rates with the TM@CeO_2_ NPs are approximately 1.6 times faster than that with CeO_2_ NPs. We speculate that this is due to the enhanced photocatalytic performance of the TM@CeO_2_ NPs compared to that of the CeO_2_ NPs. As mentioned above, HMF, which contains aldehyde and alcohol groups, is converted to FDCA via pathways that produce HMFCA and/or DFF (Scheme 1). To trace the exact reaction mechanism of the photolysis of HMF, we performed a quantitative analysis of the HMFCA, DFF, and FDCA products formed over time using HPLC-MS during the photolysis of HMF with CeO_2_, Cr@CeO_2_, and Fe@CeO_2_ NPs, dividing it into two distinct phases as a function of the reaction time (Figure 3b). As shown, in Phase A (0–6 h) only HMFCA is produced from the selective oxidation of HMF in all three NPs. Interestingly, DFF is not produced, which clearly suggests that only the aldehyde functional group within HMF is preferentially oxidized to the carboxyl functional group. From these results, we confirmed that only HMFCA, a suitable alternative to furanic polyesters obtained from fossil resources [19,20,21], is selectively produced from HMF. In Phase B (6–24 h), FDCA starts to form gradually over time with simultaneous decreases in the concentrations of HMF and HMFCA. This definitely demonstrates that the conversion reaction from HMF to FDCA occurs only through the formation of HMFCA and not through that of DFF, suggesting that selective extraction of HMFCA from HMF is possible. Furthermore, from the initial concentration of 25 mM HMF, 9.8 and 10.1 mM FDCA were obtained over 24 h for Cr@CeO_2_ and Fe@CeO_2_ NPs, respectively, which corresponds to approximately a 40% conversion yield, approximately 3.7 times better efficiency than the CeO_2_ NPs (Table 2). We also conducted the repeated PCD experiments to check photocatalytic reusability (Figure S4). As shown in Figure S4, the photocatalytic activity of the NPs was sustained for five consecutive cycles, indicating that they are robust even with repetitive use. As a result, we found that FDCA, a sustainable replacement for petrochemicals [19,20,21], is produced from HMF with a high conversion yield in TM@CeO_2_ NPs prepared by the simple method of doping Cr and Fe ions into CeO_2_ NPs.

## 4. Conclusions

Cr@CeO_2_ and Fe@CeO_2_ NPs were developed by introducing a simple method of doping Cr and Fe ions into CeO_2_ NPs. Based on a thorough analysis of the structural and electronic variation in CeO_2_, Cr@CeO_2_, and Fe@CeO_2_ NPs, we confirmed that the density of *V*_o_ in the two TM@CeO_2_ NPs was greater than that in the CeO_2_ NPs, suggesting an improvement in their photocatalytic performance. To prove this, we demonstrated the photocatalytic activity of CeO_2_, Cr@CeO_2_, and Fe@CeO_2_ NPs toward HMF oxidation. As expected, the PCD rates of HMF with TM@CeO_2_ NPs were approximately 1.6 times faster than that with CeO_2_ NPs. More importantly, a high conversion efficiency of 40% to FDCA through a selective pathway to produce HMFCA was achieved from the photolysis of HMF in the presence of TM@CeO_2_ NPs, where HMFCA and FDCA are known to be possible starting materials for the production of sustainable alternatives to petrochemical substances.

## Figures and Tables

**Table 1:** Crystallite sizes and lattice constants of the cubic fluorite CeO_2_ crystal structure estimated through Pawley refinement for XRD spectra acquired from CeO_2_, Cr@CeO_2_, and Fe@CeO_2_ NPs. The error rates indicate the 95% confidence interval.

Type of NPs	Crystallite Size (nm)	a-Axis Lattice Parameter (Å)
CeO_2_	19.1 ± 1.0	5.4150 ± 0.0008
Cr@CeO_2_	18.3 ± 1.1	5.4123 ± 0.0004
Fe@CeO_2_	19.2 ± 0.5	5.4136 ± 0.0008

**Table 2:** PCD performance and FDCA conversion yields determined through the photolysis of 25 mM HMF for 24 h under UV irradiation of 365 nm wavelength in the presence of CeO_2_, Cr@CeO_2_, and Fe@CeO_2_ NPs; these are the data at 24 h in Figure 3.

Type of NPs	PCD Efficiency of HMF (C/C_0_)	Conversion Efficiency from HMF to FDCA (%)
CeO_2_	0.41 ± 0.05	10.8 ± 0.51
Cr@CeO_2_	0.08 ± 0.01	39.2 ± 0.78
Fe@CeO_2_	0.03 ± 0.01	40.4 ± 0.82

## Data Availability

Not applicable.

## Supplementary Materials

The following supporting information can be downloaded at: https://www.mdpi.com/article/10.3390/nano13010044/s1, Figure S1: HRTEM images of (a) CeO_2_, (b) Cr@CeO_2_, and (c) Fe@CeO_2_ NPs.; Figure S2: STEM-EDS data of (a) CeO_2_, (b) Cr@CeO_2_, and (c) Fe@CeO_2_ NPs.; Figure S3: UV-Vis DRS spectra of CeO_2_, Cr@CeO_2_, and Fe@CeO_2_ NPs.; Figure S4: Repeated PCD results for the change in the relative amount of HMF under UV irradiation of 365 nm wavelength in the presence of CeO_2_, Cr@CeO_2_, and Fe@CeO_2_ NPs.
