# Supplementary material for: Photocatalysis of Cr- and Fe-Doped CeO2 Nanoparticles to Selective Oxidation of 5-Hydroxymethylfurfural
Source: Nanomaterials (Basel). 2022 Dec 22;13(1):44. doi: 10.3390/nano13010044 (PMC9823449; doi:10.3390/nano13010044)
Supplement: Supplementary file 1 [file nanomaterials-13-00044-s001.zip › nanomaterials-2111232-supplementary.pdf]

Supplementary Information

# Photocatalysis of Cr- and Fe-doped $CeO_2$ nanoparticles to selective oxidation of 5-hydroxymethylfurfural

Jeong-Woo Nam,[1,†] Vy Ngoc Pham,[2,†] Jeong Min Ha,[1] Minjeong Shin,[3,*] Hangil Lee,[2,*] Young-Sang Youn[1,*]

[1]Department of Chemistry, Yeungnam University, Daehak-ro 280, Gyeongsan, Gyeongbuk 38541, Republic of Korea,

[2]Department of Chemistry, Sookmyung Women's University, Seoul 04310, Republic of Korea,

[3]School of Chemistry and Energy, Sungshin Women's University, 55, Dobong-ro 76 ga-gil, Gangbuk-gu, Seoul 01133, Republic of Korea

*Correspondence: mshin@sungshin.ac.kr (M.S.); easyscan@sookmyung.ac.kr (H.L.); ysyoun@yu.ac.kr (Y.-S.Y.); Tel.: +82-2-920-2772 (M.S.); Tel.: +82-2-710-9409 (H.L.); Tel.: +82-53-810-2357 (Y.-S.Y.)

†These authors contributed equally.

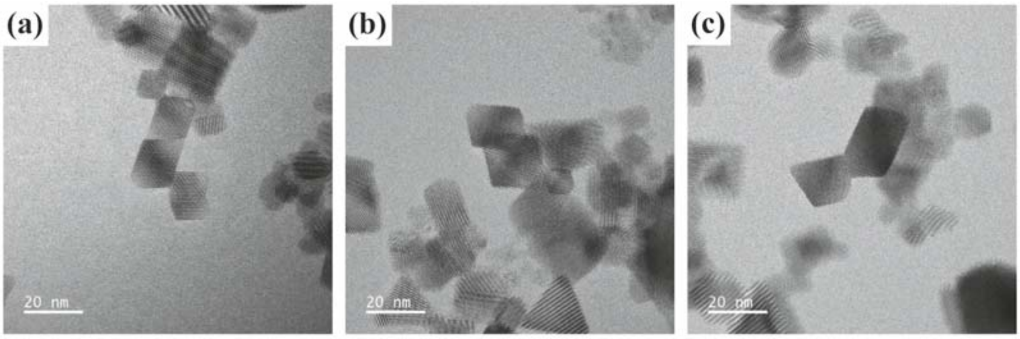


**Figure S1.** HRTEM images of (a) $CeO_2$, (b) Cr@$CeO_2$, and (c) Fe@$CeO_2$ NPs.

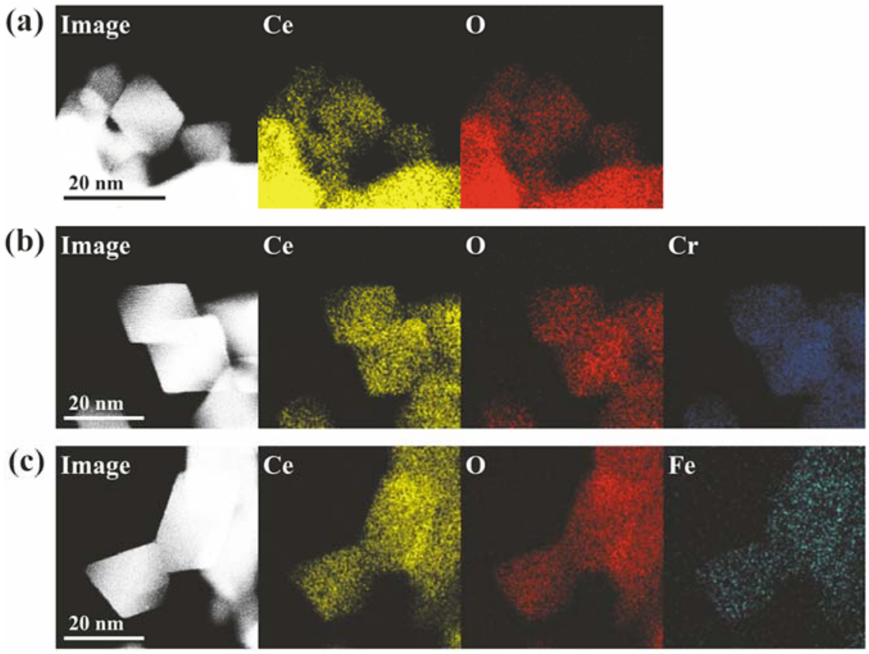


**Figure S2.** STEM-EDS data of (a) $CeO_2$, (b) Cr@$CeO_2$, and (c) Fe@$CeO_2$ NPs.

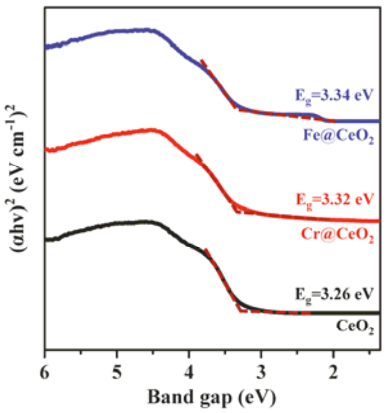


**Figure S3.** UV-Vis DRS spectra of $CeO_2$, Cr@$CeO_2$, and Fe@$CeO_2$ NPs.

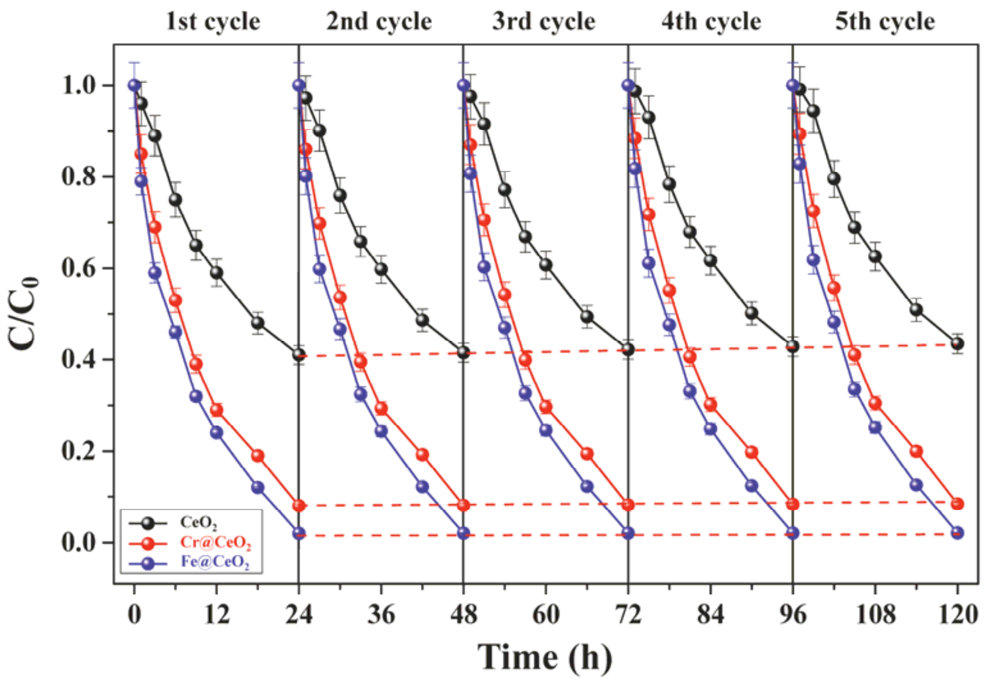


**Figure S4.** Repeated PCD results for the change in the relative amount of HMF under UV irradiation of 365 nm wavelength in the presence of $CeO_2$, Cr@$CeO_2$, and Fe@$CeO_2$ NPs, where the NPs were recovered at the end of each cycle for the reuse in the next cycle. $C_0$ and C indicate the initial concentration and post-reaction concentration of HMF, respectively.
